# Cost-effectiveness of a blended physiotherapy intervention compared to usual physiotherapy in patients with hip and/or knee osteoarthritis: a cluster randomized controlled trial

**DOI:** 10.1186/s12889-018-5975-7

**Published:** 2018-08-31

**Authors:** Corelien J. J. Kloek, Johanna M. van Dongen, Dinny H. de Bakker, Daniël Bossen, Joost Dekker, Cindy Veenhof

**Affiliations:** 1 Tranzo, Tilburg University, Tilburg, the Netherlands; 2 Netherlands Institute for Health Services Research (NIVEL), Utrecht, the Netherlands; 3 Department of Rehabilitation, Physiotherapy Sciences and Sports, Brain Center Rudolf Magnus, University Medical Center Utrecht, Utrecht, the Netherlands; 4 Expertise Center Innovation of Care, Research Group Innovation of Mobility Care, University of Applied Sciences Utrecht, Utrecht, the Netherlands; 5 Department of Health Sciences and EMGO+ Institute for Health and Care Research, Faculty of Earth and Life Sciences, VU University Medical Center Amsterdam, Amsterdam, the Netherlands; 6 ACHIEVE Centre of Expertise, Faculty of Health, Amsterdam University of Applied Sciences, Amsterdam, the Netherlands; 7 Department of Rehabilitation Medicine Amsterdam Public Health Research Institute, VU University Medical Center Amsterdam, Amsterdam, the Netherlands; 8 Department of Psychiatry Amsterdam Public Health Research Institute, VU University Medical Center Amsterdam, Amsterdam, the Netherlands

## Abstract

**Background:**

Blended physiotherapy, in which physiotherapy sessions and an online application are integrated, might support patients in taking an active role in the management of their chronic condition and may reduce disease related costs. The aim of this study was to evaluate the cost-effectiveness of a blended physiotherapy intervention (e-Exercise) compared to usual physiotherapy in patients with osteoarthritis of hip and/or knee, from the societal as well as the healthcare perspective.

**Methods:**

This economic evaluation was conducted alongside a 12-month cluster randomized controlled trial, in which 108 patients received e-Exercise, consisting of physiotherapy sessions and a web-application, and 99 patients received usual physiotherapy. Clinical outcome measures were quality-adjusted life years (QALYs) according to the EuroQol (EQ-5D-3 L), physical functioning (HOOS/KOOS) and physical activity (Actigraph Accelerometer). Costs were measured using self-reported questionnaires. Missing data were multiply imputed and bootstrapping was used to estimate statistical uncertainty.

**Results:**

Intervention costs and medication costs were significantly lower in e-Exercise compared to usual physiotherapy. Total societal costs and total healthcare costs did not significantly differ between groups. No significant differences in effectiveness were found between groups. For physical functioning and physical activity, the maximum probability of e-Exercise being cost-effective compared to usual physiotherapy was moderate (< 0.82) from both perspectives. For QALYs, the probability of e-Exercise being cost-effective compared to usual physiotherapy was 0.68/0.84 at a willingness to pay of 10,000 Euro and 0.70/0.80 at a willingness to pay of 80,000 Euro per gained QALY, from respectively the societal and the healthcare perspective.

**Conclusions:**

E-Exercise itself was significantly cheaper compared to usual physiotherapy in patients with hip and/or knee osteoarthritis, but not cost-effective from the societal- as well as healthcare perspective. The decision between both interventions can be based on the preferences of the patient and the physiotherapist.

**Trial registration:**

NTR4224 (25 October 2013).

**Electronic supplementary material:**

The online version of this article (10.1186/s12889-018-5975-7) contains supplementary material, which is available to authorized users.

## Background

Osteoarthritis (OA) is a chronic disease which mostly affects the hip and knee. People with OA experience pain, stiffness and limitations in physical functioning [1]. Worldwide, OA is the most common joint disease [2]. In the Netherlands, the prevalence is 22.5 per 1000 for hip OA and 32.2 per 1000 for knee OA [3]. In 2011, Dutch healthcare costs related to OA, including primary care, secondary care, alternative medicine and medication expenditures, were estimated to be about 1.1 billion Euros [4]. Due to the rising life expectancy and number of people with obesity, the prevalence of OA is expected to further increase during the next decades [2], which will in turn lead to an extra demand for OA-related healthcare services.

Exercise therapy as one component of physiotherapy is the most recommended conservative treatment for patients with hip and knee OA [5, 6]. Physiotherapeutic modalities like aerobic exercise, muscle strengthening and education have shown to be effective in reducing pain and improving physical functioning [7, 8]. However, face-to-face guidance is costly and the rising number of people with OA [2] requires new solutions to regulate OA-related healthcare costs. A promising strategy for reducing OA-related healthcare costs is the use of web-applications [9]. Websites and apps have the potential to partly replace face-to-face physiotherapy sessions. Next to this, websites and apps provide possibilities to support patients in taking an active role within their disease management. This new way of delivering physiotherapy, in which therapeutic guidance and an online support are integrated, is called “blended care” [10].

To the best of our knowledge, studies on the cost-effectiveness of blended interventions for patients with OA are lacking. Within mental healthcare, however, blended care for anxiety disorders, depression, smoking cessation and alcohol misuse was found to have a high probability of being cost-effective compared with wait-list, face-to-face mental healthcare, telephone counseling or unguided online care [11]. In the field of physiotherapy, a recent study showed that a blended cardiac rehabilitation intervention with minimal therapeutic guidance was cost-effective compared to center-based cardiac rehabilitation [12].

In order to investigate whether the integration of a web-application within physiotherapeutic treatment for patients with hip and/or knee OA can substitute a part of the face-to-face sessions, we developed and evaluated e-Exercise [13–15]. This blended intervention consists of a web-application integrated within regular face-to-face physiotherapy sessions. A recent cluster randomized controlled trial revealed no differences in effectiveness for physical functioning and physical activity compared to usual physiotherapy. Within group improvements in physical functioning were significant in both groups, both at the short- and long-term. Although e-Exercise was not more effective than usual physiotherapy, a difference between both groups was found in terms of the number of face-to-face sessions: i.e. the usual physiotherapy group received twelve face-to-face sessions and the e-Exercise group received five sessions [14]. It is unknown whether this reduction in face-to-face sessions also leads to a reduction of societal and/or healthcare costs and whether e-Exercise is cost-effective compared to usual physiotherapy. Therefore, the aim of this study is to evaluate the cost-effectiveness of e-Exercise compared to usual physiotherapy in patients with OA of hip and/or knee. A primary analysis was performed from the societal perspective and a secondary analysis from that of the healthcare sector.

## Methods

### Design overview

This economic evaluation was conducted alongside a 12-month prospective, single-blinded, multicenter cluster randomized controlled trial (RCT) [14, 15]. The Medical Ethical Committee of the St. Elisabeth hospital Tilburg in the Netherlands approved the study design and protocol (Dutch Trial Register NTR4224 http://www.trialregister.nl/trialreg/admin/rctview.asp?TC=4224). The trial is reported according to the CONSORT Cluster Trials checklist (Additional file 1) and the Consolidated Health Economic Evaluation Reporting Standards (CHEERS) (Additional file 2).

A total of 143 primary care physiotherapy practices from the Dutch provinces Utrecht, Noord-Holland and Gelderland with 248 eligible physiotherapists, which treated at least six OA patients per year, were randomized according to an 1:1 allocation ratio using a computer-generated sequence table. Half of the physiotherapists (*N* = 123) were instructed to treat their patients with OA of the hip and/or knee according to the e-Exercise protocol, the other half (*N* = 125) treated their patients as usual. All physiotherapists received a half-day instruction course about the study procedure. Physiotherapists allocated to e-Exercise also received an account to the website and instructions about the intervention. Physiotherapists allocated to usual physiotherapy received their e-Exercise account and instructions after the study period. Enrollment of patients lasted from September 2014 till March 2015, after which they were followed-up for 12 months.

### Participants

Patients who visited a participating physiotherapy practice were invited to participate in the study. The physiotherapist assessed eligibility, which concerned: (1) age 40–80 year, (2) OA of the hip and/or knee according to the clinical criteria of the American College of Rheumatology (ACR) [16], (3) not on a waiting list for hip or knee replacement surgery, (4) no contra-indications for physical activity without supervision according to the Physical Activity Readiness Questionnaire (PAR-Q), (5) being insufficiently physically active according to the physiotherapist (i.e. less than 30 min of moderate physical activity, 5 days per week), (6) no participation in a physiotherapy and/or physical activity program for patients with OA in the last six months, (7) access to internet, and (8) ability to understand the Dutch language. Interested patients received an information letter, an informative phone call from the main investigator (CK) and were asked to sign informed consent. Gathered patient information was stored separately from study outcomes, using an individual trial code. The main investigator (CK) was blinded to group assignment until completion of the statistical analyses.

### Intervention: E-exercise

E-Exercise is a 12-weeks intervention, in which (1) five face-to-face half-our sessions with a physiotherapist are integrated with (2) a web-application consisting of a graded activity module, exercises, and information modules. The e-Exercise intervention is based on cognitive behavioral principles and the Dutch OA guideline [17]. The physiotherapist and patient both have an e-Exercise account. Physiotherapists only needed to log in during face-to-face sessions, since there was no reimbursement for telemedicine usage. Within the physiotherapists account, the physiotherapists could adapt the online program to the patients’ individual needs and monitor patients’ log-in frequencies and assignment evaluations. The patients received automatic emails to inform and remind them about new assignments and content every week. The online graded activity module started with a baseline-measurement and formulation of a short- and long-term goal. Next, this module provided three automatically generated weekly assignments for a self-chosen activity, for example walking or cycling, gradually increased up to the personal short-term goal. The online exercise modules consisted of two weekly strength- and stability exercises selected by the physiotherapists. The online information module provided weekly new content (text and video) about an OA related topic (e.g. OA etiology, pain-management, and physical activity). Patients were asked to evaluate the execution of their assignments every week, followed by automatically generated tailored feedback. During the face-to-face physiotherapy sessions, the patients’ progress was discussed. The online e-Exercise application can be visited at https://www.e-exercise.nl [in Dutch] and a promotional video with English subtitles can be found at https://www.youtube.com/watch?v=4l9GoQWWy58.

### Intervention: usual physiotherapy

Usual physiotherapists were encouraged to treat their patients with OA according to the Dutch OA guideline, which recommends: 1) information, 2) physical exercise, and 3) strength and stability exercises [17]. No restrictions were given with regard to the number of face-to-face sessions. During the course of the study, there was no common rule on how many sessions were paid by the Dutch healthcare system. This number was dependent of the type of additional insurance of the individual patient. Some patients received no reimbursement for physiotherapy, others for examples 7, 14 or 21 sessions.

### Clinical outcome measures

Clinical outcomes for this cost-effectiveness analyses included health-related quality of life, physical functioning and physical activity. Outcomes were assessed at baseline, 3 and 12 months using online questionnaires.

1. Health-related quality of life was assessed using the EQ-5D-3 L [18]. This questionnaire differentiates 245 health states, which were converted into a utility score (0–1), based on the Dutch tariff [19]. Quality-adjusted life years (QALY’s) were calculated by multiplying patients’ utility score by their time spent in that particular health state [20].
2. Physical functioning was assessed with the subscale “function in daily living” of the Hip OA Outcome Score (HOOS) for patients with hip OA and/or the Knee Injury and OA Outcome Score (KOOS) for patients with knee OA [21, 22]. In patients with hip and knee OA, the lowest score of the HOOS and KOOS was used (0–100).
3. Physical activity was assessed with Actigraph GT3x tri-axial accelerometers. Patients were instructed to wear the accelerometer for five executive days. Data were eligible if patients wore the meter ≥3 days, for ≥8 h per day [23]. Sedentary activity, light, moderate and vigorous physical activity were distinguished according to the thresholds of Freedson et al. [24]. Moderate and vigorous physical activity were summed and translated into a score of minutes moderate and/or vigorous physical activity/day.

### Cost outcome measures

Costs included intervention, healthcare, sports, informal care, absenteeism, presenteeism, and unpaid productivity costs related to OA of hip and/or knee. Cost outcome measures were assessed at baseline, 3, 6 and 12 months using online self-reported questionnaires (Additional file 3). Since the majority of expenditures were in 2015, all costs were converted to Euros 2015, using consumer price indices [25]. Discounting was not necessary, because follow-up was limited to 1 year.

1. Intervention costs: Costs of both intervention groups consisted of the self-reported number of face-to-face physiotherapy sessions, valued by Dutch standard costs [26]. For the e-Exercise group, intervention costs also comprised development, hosting, and maintenance costs of the website, divided by the number of patients allocated to e-Exercise.
2. Healthcare costs: Patients reported their total number of physiotherapy visits after the intervention period, as well as their total number of visits to a general practitioner, massage therapist, alternative therapist, medical specialist, their hospital usage as well as their use of prescribed and over the counter drugs and medical devises during the entire study period. During data-cleaning it appeared that 16 people reported 2 or 3 hip or knee replacements within 1 year. To validate these data, all patients that reported ≥1 surgeries were contacted again in June 2017. Data derived during this contact were used for further analyses. Healthcare volumes were valued using Dutch standard costs [26], prices according to professional organizations, and unit prices of the Royal Dutch Society of Pharmacy [27].
3. Sports costs: Patients reported their sports membership costs as well as their expenses on sports equipment (e.g. shoes, clothes, racket).
4. Informal care costs: Care by family and other volunteers was valued using a recommended Dutch shadow price of 14.58 Euro /h [26].
5. Absenteeism costs: Patients were asked to report their total number of sickness absence days due to OA of hip and/or knee. In accordance with the Friction Cost Approach (friction period = 60 days), sickness absence days were valued using gender-specific price weights [28].
6. Presenteeism costs: Presenteeism was estimated using the Productivity and Disease Questionnaire (PRODISQ), valued using gender-specific price weights [26, 29–32].
7. Unpaid productivity costs: volunteer and domestic work that patients were not able to perform due to their OA was valued using a recommended Dutch shadow price of 14.58 Euro/h [26].

### Demographics

Patient characteristics, including age, sex, height, weight, educational level, location of OA, duration of OA and the presence of comorbidities, were assessed at baseline.

### Statistical analysis

Statistical analysis were performed according to the intention-to-treat principle. Descriptive statistics were used to describe and compare general characteristics of patients in the e-Exercise group and the usual physiotherapy group, and patients with complete and incomplete data. Missing data were multiply imputed (MI) in accordance with the MICE procedure [33]. We assumed that data was missing at random and specified a fully conditional model for the MI procedure. MI was stratified for treatment group but did not take into account the multilevel structure of the data. The imputation model included variables that differed between e-Exercise and usual physiotherapy at baseline, variables that were related to the “missingness” of data, variables related to the outcomes, and all available baseline and follow-up cost and effect measure values. Results of each dataset were analyzed separately as described below, and pooled according to Rubin’s rules [33].

A primary analysis was performed from the societal perspective and a secondary analysis from that of the healthcare sector. The societal perspective consisted of total costs related to osteoarthritis, irrespective of who paid for it (i.e. cost outcome measure number 1–7). The healthcare perspective included only costs accruing to the healthcare sector (i.e. cost outcome measure number 1–2).

In this trial, cluster randomization was performed. Ignoring clustering of data may lead to underestimation of uncertainty and inaccurate point estimates [34]. Therefore, differences in costs and effects between e-Exercise and usual care at 12-month follow-up were analyzed using linear multilevel analyses. Two levels were identified: patients (*n* = 208) and physiotherapists (*n* = 108). Analyses were adjusted for baseline levels of clinical outcome measures (i.e. utility score, physical functioning and physical activity), sex, BMI, level of education and location of OA. The 95%CI’s around cost differences were estimated using bias-corrected bootstrap intervals, with 5000 replications – stratified by physiotherapist. Incremental cost-effectiveness ratios (ICERs) were calculated by dividing the differences in costs between both groups by the difference in effects. Bootstrapped incremental cost-effect pairs were plotted on cost-effectiveness planes (5000 replications). Using the net monetary benefit approach [35], cost-effectiveness acceptability curves (CEACs) were constructed to provide a summary measure of the joint uncertainty of surrounding costs and effects. CEACs provide an indication of the probability of e-Exercise being cost-effective compared to usual physiotherapy at different willingness-to-pay values. For QALY’s, the probabilities were provided for a willingness-to-pay of 10,000 Euro and 80,000 Euro per patient [25]. For physical functioning and physical activity, the maximum probabilities were provided.

### Sensitivity analysis

Two sensitivity analysis were performed. The first sensitivity analysis was performed by using total costs data of complete cases with follow-up-data at each time-point that additionally completed all questionnaires. The second sensitivity analysis was a per-protocol analyses, performed by comparing total costs of patients from the e-Exercise group that completed ≥8 modules (out of 12) with the entire usual physiotherapy group.

For the cost and effect differences, a two-tailed significance level of 0.05 was considered as statistically significant. Analyses were carried out using STATA Corp 13.0 and SPSS Statistics 23.0.

## Results

### Participants

**Fig. 1:**
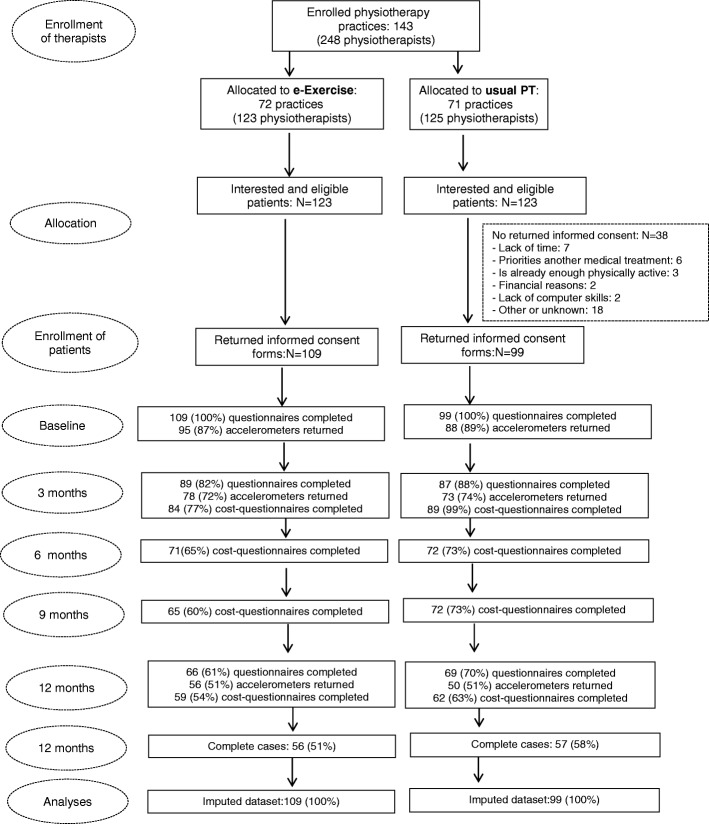
Flow chart

In total, 208 eligible patients participated in this study; 109 in the e-Exercise group and 99 in the usual physiotherapy group (Fig. 1). Patients in de e-Exercise group were recruited by 54 physiotherapists, patients in the usual physiotherapy group were recruited by 46 physiotherapists. At baseline, the e-Exercise group consisted of more low-educated people compared to the usual physiotherapy group (e-Exercise 24.8%; usual PT 12.1%; *p* = 0.04). Also, physical functioning was significantly higher in the e-Exercise group compared to the usual physiotherapy group (e-Exercise 61.3 (SD 18.3); usual physiotherapy 55.5 (SD 21.4); *p* = 0.04). Clinical outcome questionnaires were complete in 135 patients (65%), accelerometer data were complete in 106 patients (51%) and cost outcome measures were complete in 113 participants (54%). Demographics and characteristics are shown in Table 1.

**Table 1:** Baseline characteristics of e-exercise and usual physiotherapy (PT) patients

Intervention		e-Exercise	e-Exercise	e-Exercise	Usual PT	Usual PT	Usual PT
		All	Complete	Incomplete	All	Complete	Incomplete
Number of respondents		*N* = 109	*N* = 56	*N* = 53	*N* = 99	*N* = 57	*N* = 42
Sex, N (%)	Female	74 (67.9)	35 (62.5)	39 (73.6)	67 (67.7)	39	28
	Male	35 (32.1)	21 (37.5)	14 (26.4)	32 (32.3)	18	24
Age (years), mean (SD)		63.8 (8.5)	64.0 (3.9)	63.5 (10.0)	62.3 (8.9)	61.9 (8.8)	62.9 (9.2)
BMI (kg/m^2^), mean (SD)		27.8 (4.2)	27.1 (4.2)	28.5 (4.2)	27.9 (4.9)	27.7 (4.9)	28.1 (4.8)
Location OA, N (%)	Knee	71 (65.1)	37 (66.1)	34 (64.2)	67 (67.6)	38 (66.7)	29 (69.0)
	Hip	21 (19.3)	15 (26.8)	6 (11.3)	17 (17.2)	11 (19.3)	6 (14.3)
	Both	17 (15.6)	4 (7.1)	13 (24.5)	15 (15.2)	8 (14.0)	7 (16.7)
Duration of symptoms, N (%)	< 1 year	21 (19.3)	8 (14.3)	13 (24.5)	20 (20.2)	14 (24.6)	6 (14.1)
	1–5 year	42 (38.5)	28 (50.0)	14 (26.4)	38 (38.4)	23 (40.4)	15 (35.7)
	≥5 year	46 (42.2)	20 (35.7)	26 (49.1)	41 (41.4)	20 (35.1)	21 (50.0)
Education, N (%)	Low	27 (24.8)	14 (25.0)	13 (24.5)	12 (12.1)	6 (10.5)	6 (14.3)
	Middle	41 (37.6)	24 (42.9)	17 (32.1)	51 (51.5)	29 (50.9)	22 (52.4)
	High	41 (37.6)	18 (32.1)	23 (43.4)	36 (36.4)	22 (38.6)	14 (33.3)
Comorbidity, N (%)	0	62 (56.9)	29 (51.8)	33 (62.3)	62 (62.6)	31 (54.4)	31 (73.8)
	1	20 (18.3)	11 (19.6)	9 (17.0)	20 (20.2)	15 (26.3)	5 (11.9)
	≥2	27 (24.8)	16 (28.6)	11 (20.8)	17 (17.2)	11 (19.3)	6 (14.3)
Physical functioning, mean (SD)	0–100	61.3 (18.3)	64.8 (15.1)	57.6 (20.7)	55.5 (21.4)	55.9 (21.7)	55.0 (21.2)
Physical activity, mean (SD)	Min/day	25.2 (23.1)	27.3 (26.0)	22.6 (18.8)	22.5 (21.8)	25.8 (23.7)	17.1 (17.3)
Pain, mean (SD)	0–10	5.1 (2.2)	4.7 (2.1)	5.5 (2.3)	5.7 (2.3)	5.8 (2.4)	5.5 (2.1)
Utility score, mean (SD)	0–1	0.8 (0.1)	0.8 (0.1)	0.7 (0.2)	0.7 (0.2)	0.7 (0.2)	0.7 (0.2)

### Effects

At 12 months, no significant differences were seen between the e-Exercise group and the usual physiotherapy group on health-related quality of life (ΔE = 0.01; 95%CI: -0.03 to 0.04), physical functioning (ΔE = 1.49; 95%CI: -4.70 to 7.69) and physical activity (ΔE = − 3.46; 95%CI: -11.66 to 4.73).

### Resource use and costs

Patients in the e-Exercise group reported to have had on average 5 face-to-face physiotherapy sessions, whereas patients in the usual physiotherapy group reported to have had on average 12 face-to-face sessions. Consequently, intervention costs of e-Exercise were significantly lower compared to usual physiotherapy. Medication costs and sports costs were also significantly lower in the e-Exercise group compared to the usual physiotherapy group. Primary healthcare costs, secondary healthcare costs, informal care costs, absenteeism costs, presenteeism costs and unpaid productivity costs did not significantly differ between groups. Overall, total societal costs and total healthcare costs showed no statistical significant differences between groups (Table 2). A detailed overview of sub-categories of healthcare costs in complete cases are provided in Additional file 4.

**Table 2:** Mean costs per participant in the e-Exercise group and usual physiotherapy (PT) group and mean differences between both groups during 12 months follow-up

Cost category	e-Exercise (*N* = 109); mean costs in € (SEM)	Usual PT (*N* = 99); mean costs in € (SEM)	Unadjusted mean cost difference in € (95% CI)	Adjusted mean cost difference in € (95% CI)
Intervention ^a^	241 (37)	451 (55)	− 209 (− 294 to − 128)	− 202 (− 286 to − 120)
Primary healthcare	438 (63)	536 (84)	−98 (− 306 to 80)	− 107 (− 340 to 82)
Secondary healthcare	3143 (711)	3819 (885)	− 677 (− 2699 to 1138)	− 332 (− 2134 to 1444)
Medication ^a^	106 (24)	299 (90)	− 192 (− 436 to − 79)	− 151 (− 340 to −52)
Sport	159 (26)	292 (73)	− 133 (− 242 to −51)	− 126 (− 237 to − 43)
Informal care	327 (109)	327 (80)	1 (− 173 to 156)	46 (− 117 to 205)
Absenteeism	927 (434)	743 (304)	184 (−64 to 1092)	368 (− 459 to 1365)
Presenteeism	237 (74)	429 (121)	− 191 (− 533 to 12)	−120 (− 411 to 64)
Unpaid productivity	768 (137)	823 (162)	−55 (− 397 to 256)	97 (− 219 to 413)
Healthcare costs^b^	3928 (744)	5105 (937)	− 1177 (− 3340 to 763)	− 792 (− 2720 to 1100)
Total costs	6348 (1007)	7718 (1292)	− 1371 (− 4512 to 1240)	− 529 (− 3315 to 2057)

^a^Significant difference between e-Exercise and usual PT

^b^Healthcare costs = intervention costs + primary healthcare costs + secondary healthcare costs + medication costs

Adjusted for sex, age, BMI, level of education, type of OA, duration of OA, physical functioning at baseline, pain at baseline and utility score at baseline

### Cost-effectiveness analysis

#### Primary analysis: Societal perspective (total costs)

**Fig. 2:**
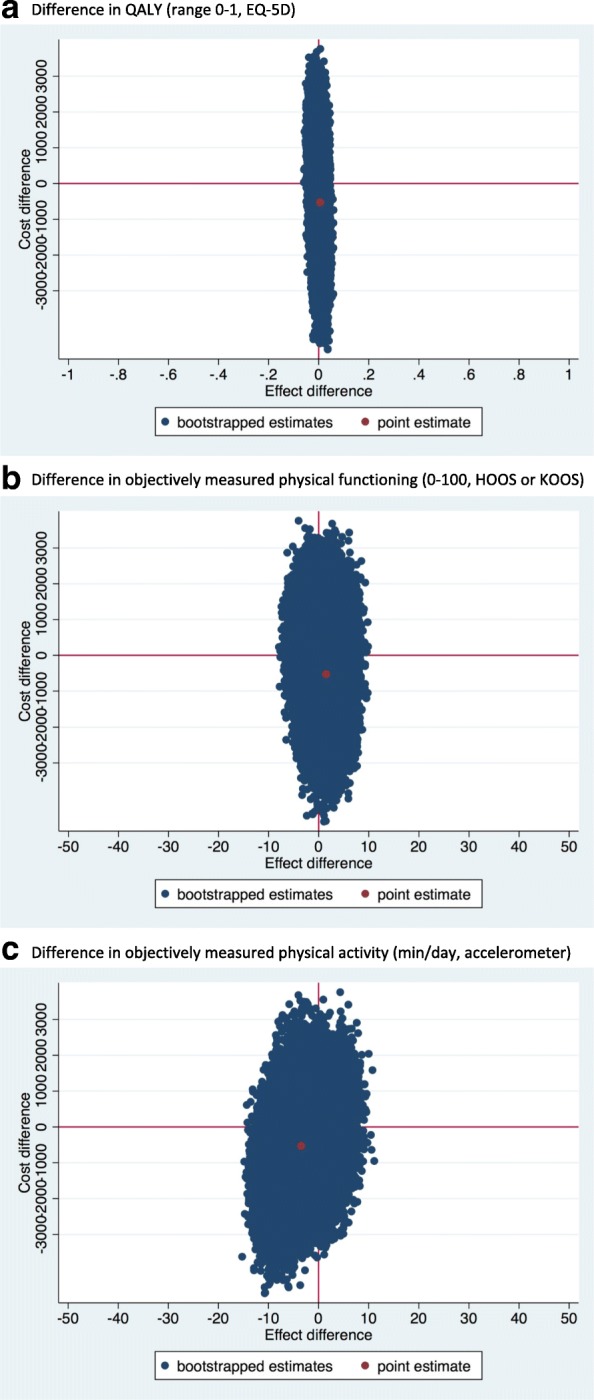
Cost effectiveness planes from societal perspective. **a** Difference in QALY (range 0-1, EQ-5D). **b** Difference in physical functioning (0-100, HOOS or KOOS). **c** Difference in objectively measured physical activity (min/day, accelerometer)

**Fig. 3:**
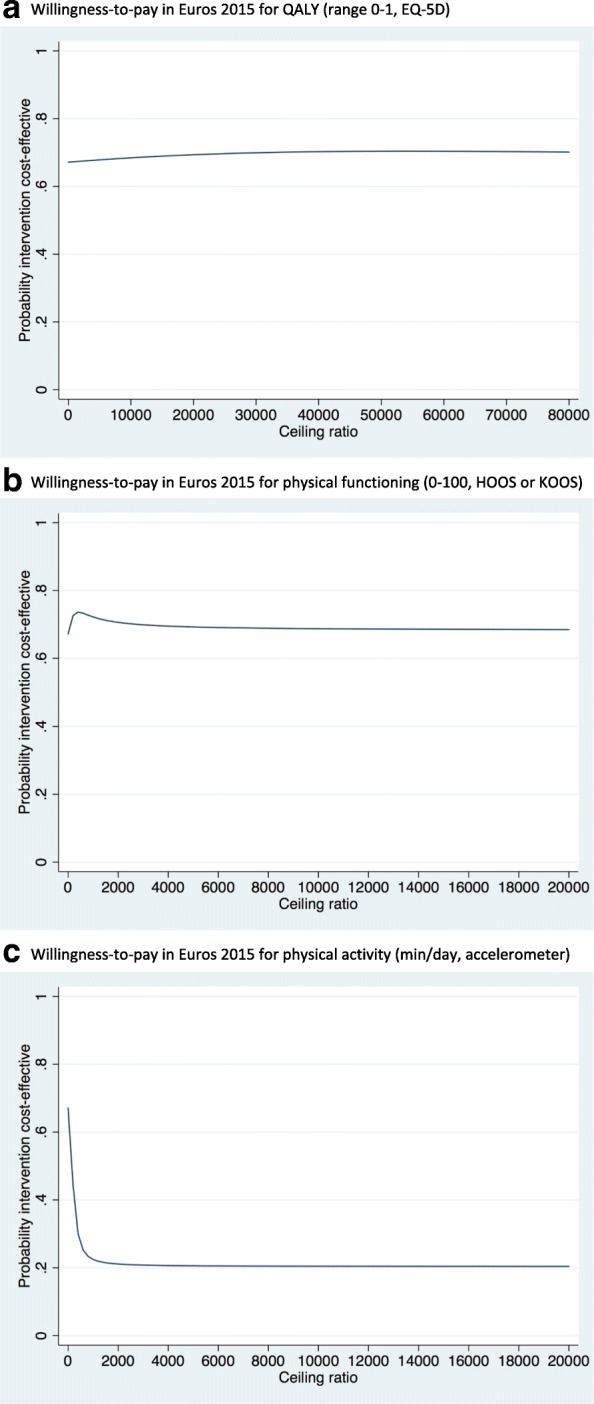
Cost-effectiveness acceptability curves from societal perspective. **a** Willingness-to-pay in Euros 2015 for QALY (range 0-1, EQ-5D). **b** Willingness-to-pay in Euros 2015 for physical functioning (0-100, HOOS or KOOS). **c** Willingness-to-pay in Euros 2015 for physical activity (min/day, accelerometer)

For QALYs, the ICER was − 52,900 Euro/point (i.e.-529/0.01), demonstrating that one QALY gained in e-Exercise was on average associated with a societal cost saving of 52,900 Euro compared to usual physiotherapy indicating that e-Exercise was dominant over usual physiotherapy (Table 3, Fig. 2a). However, as shown by the scatterplot (Fig. 2a), the CEAC (Fig. 3a) and the quadrants of the CE plane (Table 3), uncertainty was large and the probability of e-Exercise being cost-effective compared to usual physiotherapy was 0.68 at a willingness to pay of 10,000 Euro per QALY gained and 0.70 at a willingness to pay of 80,000 Euro per QALY gained.

**Table 3:** Differences in pooled mean costs and effects

Analysis	N e-Exercise	N Usual PT	Outcome	ΔC (95% CI) In euro’s	ΔE (95% CI) In points	ICER Euro/point	Distribution CE-plane (%)			
							NE^a^	SE^b^	SW^c^	NW^d^
Main analysis 1:	109	99	QALYs (0–1)	−529 (− 2265 to 1268)	0.01 (− 0.03 to 0.04)	−52,900	17.8	42.1	23.2	16.9
*Total costs and imputed dataset*	109	99	Physical functioning (0–100)	−529 (−2265 to 1268)	1.49 (−4.70 to 7.69)	−355	20.5	44.7	20.6	14.2
	109	99	Physical activity (min/day)	−529 (−2265 to 1268)	−3.46 (−11.66 to 4.73)	153	7.7	9.4	55.9	27.0
Main analysis 2:	109	99	QALYs (0–1)	−792 (− 2101 to 440)	0.01 (−0.03 to 0.04)	−79,200	13.5	46.4	33.4	6.7
*Healthcare costs and imputed dataset*	109	99	Physical functioning (0–100)	−792 (−2101 to 440)	1.49 (−4.70 to 7.69)	−532	14.4	50.7	29.2	5.7
	109	99	Physical activity (min/day)	−792 (−2101 to 440)	−3.46 (−11.66 to 4.73)	229	5.2	11.9	67.9	15.0
Sensitivity analysis 1:	42	36	QALYs (0–1)	2211 (701 to 3722)	−0.00 (−0.03 to 0.03)	−22,110	34.8	0.1	0.1	65.0
*Complete cases*	42	36	Physical functioning (0–100)	2211 (701 to 3722)	−2.15 (−7.50 to 3.20)	−1.028	18.4	0.0	0.2	81.4
	42	36	Physical activity (min/day)	2211 (701 to 3722)	−1.95 (−7.43 to 3.53)	− 1.134	22.0	0.0	0.2	77.8
Sensitivity analysis 2:	39	99	QALYs (0–1)	− 592 (− 2719 to 1603)	0.02 (−0.01 to 0.06)	− 29,600	29.0	65.6	1.4	4.0
*Per-protocol and imputed dataset*	39	99	Physical functioning (0–100)	−592 (−2719 to 1603)	4.10 (−1.56 to 9.77)	− 144	30.7	66.1	0.8	2.4
	39	99	Physical activity (min/day)	−592 (−2719 to 1603)	−1.79 (− 8.72 to 5.13)	331	12.8	18.3	48.7	20.2

*CI* confidence interval, *C* costs, *CE-plane* cost-effectiveness plane, *E* effects, *ICER* incremental cost-effectiveness *Ratio* costs are expressed in 2015 Euros

^a^The northeast quadrant of the CE plane, indicating that e-Exercise is more effective and more costly than usual physiotherapy

^b^The southeast quadrant of the CE plane, indicating that e-Exercise is more effective and less costly than usual physiotherapy

^c^The northwest quadrant of the CE plane, indicating that e-Exercise is less effective and more costly than usual physiotherapy

^d^The southwest quadrant of the CE plane, indicating that e-Exercise is less effective and less costly than usual physiotherapy

For physical functioning, the point estimate of the ICER was − 355 Euro/point (i.e. -529/1.49), indicating dominance of e-Exercise compared to usual physiotherapy (Table 3, Fig. 2b). The CEAC (Fig. 3b) showed that if decision makers are not willing to pay anything per 1-point improvement on the HOOS/KOOS, the probability of e-Exercise being cost-effective compared to usual physiotherapy was 0.67. At higher willingness to pays, this probability remained about the same.

For physical activity, the point estimate of the ICER was 153 Euro/point (− 529/− 3.46), indicating dominance of usual physiotherapy compared to e-Exercise (Table 3, Fig. 2c). The CEAC (Fig. 3c) showed that if decision makers are not willing to pay anything per 1-min improvement of physical activity per day, the probability of e-Exercise being cost-effective compared to usual physiotherapy was 0.67. At higher willingness to pays, this probability decreased.

Overall, from the societal perspective, the maximum probability of e-Exercise being cost-effective compared with usual physical therapy was moderate.

#### Secondary analysis: Healthcare perspective (healthcare costs)

The ICER for QALYs was − 79,200 Euro/point (i.e. -792/0.01), indicating dominance of e-Exercise compared to usual physiotherapy (Table 3). However, the CEAC (not shown) indicated large uncertainty and the probability of cost-effectiveness was 0.84 at a willingness to pay of 10,000 Euro per QALY gained and 0.80 at a willingness to pay of 80,000 Euro per QALY gained.

The point estimate of the ICER for physical functioning was − 532 Euro/point (i.e. -792/1.49), indicating dominance of e-Exercise compared to usual physiotherapy (Table 3). The CEAC (not shown) showed that if decision makers are not willing to pay anything per 1-point improvement on the HOOS/KOOS, the probability of cost-effectiveness was 0.82. At higher willingness to pays, this probability remained about the same.

For physical activity, the point estimate of the ICER was 229 Euro/point (i.e. -792/− 3.46), indicating dominance of usual physiotherapy compared to e-Exercise (Table 3). The CEAC (not shown) showed that if decision makers are not willing to pay anything per 1-min improvement of physical activity per day, the probability of cost-effectiveness was 0.82. At higher willingness to pays, this probability remained about the same.

Overall, from the healthcare perspective, the maximum probability of e-Exercise being cost-effective compared with usual physical therapy was moderate.

### Sensitivity analysis

Results of the sensitivity analyses with complete-cases showed significant higher costs in the e-Exercise group compared to usual physiotherapy, but no significant differences in effects. Results of the per-protocol sensitivity analysis were in line with those of the main analysis (Table 3).

## Discussion

### Main findings

This study showed that the intervention costs of e-Exercise were significantly lower compared to usual physiotherapy in patients with hip and/or knee OA due to the fact that e-Exercise patients received on average seven face-to-face sessions less than their usual physiotherapy counterparts. Medication costs were also significantly lower in e-Exercise compared to usual physiotherapy, whereas total societal and total healthcare costs did not significantly differ between groups. There were small differences in both societal and healthcare costs in favor of the e-Exercise arm, however differences were not proven significant. Thus, e-Exercise was dominant as compared to usual physiotherapy. Moreover, we consider the probabilities of e-Exercise being cost-effective compared with usual physiotherapy to be low at the lower and upper bound of the Dutch willingness to pay threshold for QALYs (i.e. 0.68 at 10,000 Euro/QALY and 0.70 at 80,000 Euro/QALY). For all other outcomes, willingness to pay thresholds are lacking, but we consider the associated maximum probabilities of e-Exercise being cost-effective compared to usual physiotherapy to only be moderate (i.e. < 0.82). Therefore, we do not consider the intervention cost-effective as compared to usual care. These results were confirmed by a per-protocol sensitivity analysis. However, the sensitivity analysis using complete cases only showed significant higher costs in the e-Exercise group compared to the usual physiotherapy group. The latter is probably due to selective drop-out. That is, patients with complete and incomplete data slightly differed in terms of levels of physical functioning and physical activity, with higher levels of physical functioning and physical activity at baseline in patients with complete data. This indication of selective drop out suggests that the results of the main analysis (for which data us imputed) are more valid than those of the sensitivity analysis (for which only complete cases were used).

### Interpretation of the findings

An explanation for the absence of a significant difference in total societal and healthcare costs between e-Exercise and usual physiotherapy might be the fact that physiotherapeutic sessions are relatively cheap compared to for example secondary healthcare costs, like outpatient clinic visits. Hence, intervention cost comprises only a small share of the total societal and healthcare costs. Within economic evaluations it is warranted to include all relevant cost categories, instead of only including intervention costs [26]. Although we did find significantly lower intervention and medication costs within e-Exercise, the share of these cost categories is relatively small (interventions costs 4% of total costs; medication: 2% of total costs) compared to that of secondary healthcare costs (50% of total costs). Taken all healthcare costs together, total costs were still in favor of e-Exercise, albeit not statistically significantly. One should bear in mind, however, that a 12-month follow-up is likely to be too short to investigate whether one of both interventions results in a reduction of secondary healthcare costs at the long-term (e.g. due to joint replacements). Therefore, for future cost-effectiveness analyses of physiotherapeutic interventions, it is recommended to use longer follow-up periods. With respect to this this study, we recommend to investigate the number of hip and/or knee replacements in both groups 5 years after baseline.

Another explanation for the finding that e-Exercise was not cost-effective compared with usual physiotherapy might be the fact that differences in effectiveness between both interventions were minimal. For physical activity, this can be explained by the fact that patients already had a high level of physical activity per day at baseline, which resulted in less room for improvement in both groups. Next to this, two mixed-methods studies provided recommendation to improve the effectiveness of e-Exercise [36, 37]. Two concrete recommendations were to provide options to tailor the intervention more to individual patient needs and to learn physiotherapists to integrate online care within physiotherapeutic care. Also, knowledge about patients that are more or less suitable for receiving a blended intervention is warranted. Improving the intervention as a whole (i.e. the web-application, the integration within physiotherapeutic care and providing it to the right person) might improve the effectiveness, as well as the cost-effectiveness of e-Exercise. Currently, e-Exercise and usual physiotherapy do not show clinically relevant, nor statistically significant differences in effectiveness, with significantly lower intervention costs in e-Exercise.

Since from both perspectives, no significant differences were seen in total costs and effects, the decision about which intervention should be applied can be based on the preferences of the patient and the physiotherapist. In the current Dutch healthcare system, however, physiotherapists get paid per session and have no financial incentive to apply an intervention with less face-to-face sessions. Physiotherapists that used e-Exercise, mentioned this financial (dis-)advantage as one of the determinants for not using e-Exercise [37]. In order to stimulate the usage of e-Exercise by physiotherapists, the investigation of new business models (like a shared-savings model or bundled payment system in which physiotherapists will receive a fixed amount of money per patient with OA, instead of getting paid per session) is recommended.

### Strengths & limitations

A strength of this economic evaluation is that we analyzed the data both from the societal perspective as well as the healthcare perspective. Next, we not only used QALYs as outcome measure, but also physical functioning and physical activity. These two outcome measures are closely related to the aim of the studied interventions. In accordance with the recommendations of the latest systematic review on economic evaluation of non-pharmacologic interventions in OA, a generic instrument for QALY calculation was used, physiotherapeutic usual care was used as comparator and follow-up lasted 1 year [38]. A limiting factor within the current study was the use of self-reported questionnaires, which were sent every 3 months. Self-reported questionnaires are a potential source of “social desirability” and/or “recall bias”. To illustrate, after analyzing the data, it appeared that 16 participants reported multiple hip and/or knee surgeries, whereas it highly unlikely for patients is to have had more than one joint replacement in 1 year. To validate these data, in June 2017 all patients that reported ≥1 surgeries were sent again the question how often they received a hip or knee replacement over the past 12 months. Although we instructed patients to only report expenditures and absenteeism related to their OA, patients might have reported costs related to other diagnoses as well. Total annual costs in this study were more than 3000 Euro per individual higher as compared to a recently published study in Dutch patients with OA of the hip [39]. The difference between the study of Tan et al. (2016) and this study is that we included patients in the physiotherapy practice instead of the general practice. Next, this study was based on a sample with both hip and knee OA. Since our power calculation was based on a sample both hip and knee OA (or a combination), sub-group analyses resulted in under-powered small groups which were too small to produce conclusive results. A final limitation is the relatively high percentage of patients with missing data, despite up to three reminders by mail and phone per questionnaire. Possibly, we might have overloaded the participants with too many measurements. As a solution, missing costs and effects were multiply imputed. Within economic evaluations, multiple imputation is a widely used method which is considered as highly appropriate since the use of several imputed data sets makes it possible to account for the uncertainty about missing data [33]. Nonetheless, a 100% complete dataset would have produced more valid and reliable results. Therefore, the current findings should be treated with caution and extensive efforts ought to be made in future studies to reduce the amount of missing data.

## Conclusion

Overall, e-Exercise cannot be seen as cost-effective in comparison with usual physiotherapy, from both a societal and a healthcare perspective. From both perspectives, no significant differences were seen in total costs and effects. Therefore, the decision about which intervention should be applied can be based on the preferences of the patient and the physiotherapist. Future research exploring which patients are more or less suitable for blended physiotherapy is warranted.

## Additional files

Additional file 1: CONSORT Cluster Randomized Trial checklist. (DOCX 34 kb)

Additional file 2: CHEERS Checklist. (PDF 571 kb)

Additional file 3: Cost questionnaire (translation of Dutch version). (DOC 70 kb)

Additional file 4: Mean costs per participant in the e-Exercise group and usual physiotherapy (PT) group during 12 months follow-up (cases with complete cost data). (DOCX 15 kb)

## Abbreviations

CEAC: Cost-effectiveness acceptability curves
HOOS: Hip osteoarthritis outcome score
ICER: Incremental cost-effectiveness ratios
KOOS: Knee osteoarthritis outcome score
OA: Osteoarthritis
QALY: Quality adjusted life years
RCT: Randomized controlled trial
