# Supplementary material for: Cost-effectiveness of a blended physiotherapy intervention compared to usual physiotherapy in patients with hip and/or knee osteoarthritis: a cluster randomized controlled trial
Source: BMC Public Health. 2018 Aug 31;18:1082. doi: 10.1186/s12889-018-5975-7 (PMC6119267; doi:10.1186/s12889-018-5975-7)
Supplement: Supplementary file 3 — Cost questionnaire (translation of Dutch version). (DOC 70 kb) [file 12889_2018_5975_MOESM3_ESM.doc]

**Additional file 3 Cost questionnaire (translation of Dutch version)**

|  | | **In the last 3 months** | |  | |
| --- | --- | --- | --- | --- | --- |
| General practitioner How many times have you been to the general practitioner because of your osteoarthritis of hip and/or knee?  How many telephonic consultations have you had because of your osteoarthritis of hip and/or knee?  How many times have your general practitioner visited you at home for your osteoarthritis of hip and/or knee? | | Number of visits  . . . . . . . . . . . . .  . . . . . . . . . . . . .  .  . . . . . . . . . . . . . | |  | |
| Paramedical treatment How many times have you visited one of these therapists because of your osteoarthritis of hip and/or knee?  Physiotherapist  Manual therapist  Occupational therapist  Exercise therapist  Other, i.e.……………………………………..  Other, i.e.…………………………………….. | | Number of visits  . . . . . . . . . . . . .  . . . . . . . . . . . . .  . . . . . . . . . . . . .  . . . . . . . . . . . . .  . . . . . . . . . . . . .  . . . . . . . . . . . . . | |  | |
| **Alternative medicine**  How many times have you visited one of these alternative therapists because of your osteoarthritis of hip and/or knee?  Chiropractor  Acupuncturist  Homeopath  Magnetizer  Other, i.e.……………………………………..  Other, i.e.…………………………………….. | | Number of visits  . . . . . . . . . . . . .  . . . . . . . . . . . . .  . . . . . . . . . . . . .  . . . . . . . . . . . . .  . . . . . . . . . . . . .  . . . . . . . . . . . . . | | Total amount of costs € . . . . . . .  € . . . . . . .  € . . . . . …  € . . . . . . .  € . . . . . . .  € . . . . . . | |
| Medical specialists How many times have you visited one of these physicians because of your osteoarthritis of hip and/or knee?  Orthopedic surgeon  General surgeon  Reumatologist  Neurological surgeon  Other, i.e.…………………………………….. | | Number of visits  . . . . . . . . . . . . .  . . . . . . . . . . . . .  . . . . . . . . . . . . .  . . . . . . . . . . . . .  . . . . . . . . . . . . . | |  | |
| Diagnostics Have you had one of these diagnostics?  X-ray  CT-scan  MRI-scan  Other, i.e.…………………………………….. | | Number of diagnostics . . . . . . . . . . . . .  . . . . . . . . . . . . .  . . . . . . . . . . . . .  . . . . . . . . . . . . . | |  | |
| Hospital visits How many days have you stayed in the hospital for your osteoarthritis of hip and/or knee? (including intensive care)  How many days have you stayed at the intensive care department for your osteoarthritis of hip and/or knee?  Have you had surgery for your osteoarthritis of you hip and/or knee? | | Number of days  . . . . . . . . . . . . .  . . . . . . . . . . . . .  Yes/No | |  | |
| Have you used medication or medical tools, as recommended by your general practitioner or physician for your osteoarthritis of hip and/or knee in the past 3 months?Prescribed medication for osteoarthritis of hip/knee Type of medication  . . . . . . . . . . . . . . . . . . . . . .  . . . . . . . . . . . . . . . . . . . . . .  . . . . . . . . . . . . . . . . . . . . . .  **Medical tools, recommended by your general practitioner or physician**  Crutches  Rollator  Other, i.e.……………………………………..  Other, i.e.…………………………………….. | Type Tablet, salve, other i.e.….  . . . . . . . . . . . . .  . . . . . . . . . . . . .  . . . . . . . . . . . . . | | Dosis . . . . . . . . .  . . . . . . . . .  . . . . . . . . . | Duration From… till…  From… till…  From… till…  From… till…  From… till…  From… till…  From… till… |  |
| Have you used medication without prescription for your osteoarthritis of hip and/or knee in the past 3 months? **Medication without prescription**  **(i.e. ibuprofen)**  Type of medication  . . . . . . . . . . . . . . . . . . . . . .  . . . . . . . . . . . . . . . . . . . . . .  . . . . . . . . . . . . . . . . . . . . . .  **Medical tools without prescription**  Brace  Bandage  Other, i.e.…………………………………….. | Type . . . . . . . . . . . . .  . . . . . . . . . . . . .  . . . . . . . . . . . . .  **Costs**    € . . . . . . .  € . . . . . . .  € . . . . . . . | | Dosis . . . . . . . . . .    . . . . . . . . . .  . . . . . . . . . . |  |  |

| **Have you had sport expenditures for your osteoarthritis of hip and/or knee in the past three months?**  Swimming tickets/contribution  Gym (contribution for 3 months)  Sport club (contribution for 3 months)  Sport equipment (for example shoes, clothes, racket)  Other, i.e.……………………………………..  Other, i.e.……………………………………..  Other, i.e.……………………………………..  Other, i.e.……………………………………..  Other, i.e.…………………………………….. | **Costs**  € . . . . . . .  € . . . . . . .  € . . . . . . .  € . . . . . . .  € . . . . . . .  € . . . . . . .  € . . . . . . .  € . . . . . . . |  |  |
| --- | --- | --- | --- |
|  |  |  |  |
| **Paid work**  How many days have you been sick at home due to your osteoarthritis of hip and/or knee?  How many days have you been to work, while feeling disabled due to your osteoarthritis of hip and/or knee (don’t include the days that you have been sick at home).  (score 1 or higher)  On a scale from 0 till 10, how many work have you done on the days that you have been to work while feeling disabled due to your osteoarthritis of hip and/or knee? 0 stands for “nothing”, 10 stands for “as much as usual”.  On a scale from 0 till 10, how do you rate the quality of your performed work on the days that you have been to work while feeling disabled due to your osteoarthritis of hip and/or knee? 0 stands for “worst possible quality”, 10 stands for “same quality as usual”.  On a scale from 0 till 10, how do you rate your general performance at work? 0 stands for “worst possible performance quality”, 10 stands for “best possible performance”. | Number of days  . . . . . . . .  . . . . . . . .  (0: nothing-10:as much as usual)  . . . . . . . .  (0: worst possible quality-10:same quality as usual)  . . . . . . . .  (0: worst possible performance 10: best possible performance)  . . . . . . . . |  |  |
| **Unpaid work**  How many hours weren’t you able to do normal unpaid activities due to your osteoarthritis of hip and/or knee:   - Domestic work, work around the house - Voluntary work - Doing groceries   Did someone replace you due to your osteoarthritis of hip and/or knee?   - Family care or home care - Family, friences, volunteers - Paid cleaner or handyman | Number of hours  . . . . . . . . . .  . . . . . . . . . .  . . . . . . . . . .  . . . . . . . . . .  . . . . . . . . . .  . . . . . . . . . . |  |  |
