# Supplementary material for: Cost-effectiveness of a blended physiotherapy intervention compared to usual physiotherapy in patients with hip and/or knee osteoarthritis: a cluster randomized controlled trial
Source: BMC Public Health. 2018 Aug 31;18:1082. doi: 10.1186/s12889-018-5975-7 (PMC6119267; doi:10.1186/s12889-018-5975-7)
Supplement: Supplementary file 4 — Mean costs per participant in the e-Exercise group and usual physiotherapy (PT) group during 12 months follow-up (cases with complete cost data). (DOCX 15 kb) [file 12889_2018_5975_MOESM4_ESM.docx]

**Additional file 4 Mean costs per participant in the e-Exercise group and usual physiotherapy (PT) group during 12 months follow-up (cases with complete cost data)**

| Cost Category | e-Exercise (N=56)  mean costs in € (SEM) | Usual Physiotherapy (N=57)  mean costs in € (SEM) |
| --- | --- | --- |
| Intervention | 212 (28) | 408 (43) |
| Primary healthcare |  |  |
| -General practitioner | 46 (10) | 48 (9) |
| -Paramedical treatment | 150 (43) | 361 (99) |
| -Alternative medicine | 84 (38) | 39 (22) |
| Secondary healthcare |  |  |
| -Visit to a medical specialist | 183 (43) | 178 (44) |
| -Diagnostic (i.e. X-ray, MRI) | 186 (42) | 183 (35) |
| -Operation and hospital stay | 1735 (723) | 1102 (407) |
| Medication |  |  |
| -Pills | 56 (18) | 244 (118) |
| -Medical tools | 13 (4) | 26 (10) |
| Sport | 129 (26) | 235 (48) |
| Informal care | 134 (60) | 465 (129) |
| Absenteeism | 226 (226) | 155 (119) |
| Presenteeism | 193 (90) | 235 (81) |
| Unpaid productivity | 416 (120) | 675 (148) |
